# The mTOR pathway is involved in the process of platelet-rich plasma improving intervertebral disc degeneration

**DOI:** 10.22038/ijbms.2024.79218.17163

**Published:** 2025

**Authors:** Jing Luan, Qi Wang, Wei Zheng, Yongjin He

**Affiliations:** 1 Department of Pain, Tianjin First Central Hospital, Tianjin, 300110, China; 2 Department of Anesthesiology, Tianjin First Central Hospital, Tianjin, 300110, China

**Keywords:** Akt/mTOR/p70S6K-signaling pathway, Apoptosis, Extracellular matrix- degradation, Inflammatory factors Intervertebral disc- degeneration, Oxidative stress, Platelet-rich plasma

## Abstract

**Objective(s):**

Platelet-rich plasma (PRP) contains multiple growth hormones that may stimulate tissue repair. We aimed to assess PRP’s efficacy and explore possible mechanisms using the intervertebral disc degeneration (IDD) model.

**Materials and Methods:**

A total of 48 male Sprague-Dawley (SD) rats were randomly divided into three groups: sham, IDD+PBS, and IDD+PRP (n=16, respectively). IL-1β (10 ng/ml) was used to establish a humanized IDD model in human lumbar nucleus pulposus (NP) tissues from 36 patients with degenerative disc disease. These NP cells were randomly divided into three groups: sham, IDD+PBS, and IDD+PRP (n=12, respectively). RT-PCR and western blot were used to detect the expression of aggrecan, collagen II, IL-1β, IL-6, TNF-α, Bcl-2, cleaved-Caspase 3, Bax and Akt/mTOR/p70S6K signaling pathway. A related assay kit was used to detect MDA, SOD, and GSH.

**Results:**

PRP affected the expression of aggrecan, collagen II, IL-1β, IL-6, TNF-α, MDA, SOD, GSH, Bcl-2, cleaved-Caspase 3, and Bax in IDD rats. Compared with the IDD+PBS group, the expression of *p-mTOR, p-p70/S6K*, and *p-Akt* was much lower in the rat IDD+PRP group (*P<*0.05). Similarly, with PRP treatment in the humanized IDD model, the expression of *p-mTOR, p-p70/S6K*, and *p-Akt* was also inhibited.

**Conclusion:**

PRP may be a potential therapy for IDD via the mTOR signaling pathway in regulating and affecting extracellular matrix degradation, inflammatory factors, oxidative stress, and apoptosis.

## Introduction

Low back pain (LBP) is a common symptom in all age groups, from children to older adults (1). Global Burden of Disease Study 2017 demonstrated that LBP was the leading cause of years lived with disability (2). It places a heavy financial burden and social problems on individuals and society (3). Intervertebral disc degeneration (IDD) has been recognized as the cause of LBP (4). Current IDD treatments include bed rest, physical therapy, and surgical techniques such as disc replacement, disc fusion, and so on (5). Bed rest and physical therapy can only relieve symptoms. Due to biomechanical changes, surgical treatment may lead to potential complications such as loss of function. As an avascular tissue, the intervertebral disc has a poor ability to heal spontaneously; oral drugs are usually ineffective.

The previous study found that inflammatory mediators and signaling pathways are involved in the development and progression of disc degeneration (6). Especially, IL-1β and TNF-α play an important role. They can induce the expression of some pain-related factors (e.g., cyclooxygenase 2, nitric oxide, and nerve growth factors), promote nerve ingrowth, and cause LBP. The development of IDD is also characterized by changes in the intervertebral disc microenvironment’s cells and extracellular matrix (ECM), resulting in progressive functional decline and structural damage. Produced by cells, ECM is a complex, highly dynamic structure that is a mechanically supportive tissue of cells and a site for supplying nutrients and immune responses. The main components of the ECM are aggrecan and collagen II, and their degradation is related to matrix metalloproteinases (MMPs) (7). Disturbed ECM metabolism may be an intermediate process in the pathomechanism of IDD (8). Oxidative stress can accelerate intervertebral disc (IVD) degeneration through multiple signaling pathways, such as the nuclear factor kappa-B (NF-κB) pathway, mitogen-activated protein kinase (MAPK) pathway, and PI3K/Akt pathway, leading to IDD (9). 

In addition to the above possible mechanisms, apoptosis and autophagy have also received increasing attention in IDD research. As classical apoptotic pathways, the death receptor, mitochondria, and endoplasmic reticulum stress (ERS) pathways have been demonstrated to be involved in the occurrence and development of IDD (10). Mitochondrial apoptosis is an important pathway in the pathogenesis of IDD. There are up-regulated Bax and down-regulated Bcl-2 in IDD. Bax could cause mitochondrial rupture by opening voltage-dependent anion channels in mitochondria, while Bcl-2 could protect normal intervertebral disc tissue from apoptosis via combining with Bax (11). The balance between Bax and Bcl-2 impacts the proapoptotic pathway. Autophagy is an evolutionary cell mechanism that could protect cells in hostile situations by degrading unnecessary intracellular components. However, excessive autophagy often leads to cell death. The mTOR pathway, a key modulator in upstream autophagy, was reported as a serine/threonine protein kinase (12). Activation of mTOR is involved in the degradation of nucleus pulposus (NP) cells (13). Up-regulated mTOR and its phosphorous were observed in the degenerated human intervertebral disc. Therefore, clarifying the role of mTOR signaling in IDD can provide a theoretical basis for the biological treatment of IDD. 

Currently, bioactive drugs have garnered significant attention due to their minimal impact on the body. Platelet-rich plasma (PRP) is an autologous blood concentrate containing high concentrations of growth factors and cytokines. Because of its ability to promote proliferation and migration, PRP is widely used to repair various avascular tissues, including bones, cartilage, muscles, and tendons (14, 15). Several *in vitro *and in vivo (animal) studies have revealed that PRP has significant biological efficacy in stimulating IVD cells to repair tissue and treat discogenic LBP caused by IDD (16). However, the mechanism is still not clear. A recent study showed that PRP induced chondroprotection via increasing autophagy, anti-inflammatory markers, and decreasing apoptosis in human osteoarthritic cartilage (17). As an upstream regulator of autophagy, mTOR plays an important role in cell growth, proliferation, and survival. The current study explored whether the mTOR pathway was involved in the process of PRP improving IDD (18, 19).

## Materials and Methods

### The annulus fibrosus needle puncture model

A total of 48 male Sprague-Dawley (SD) rats weighing 200-250 g were purchased from the Laboratory Animal Center of Nankai University. All animals were kept in a temperature-controlled room (23±2 ^°^C; relative humidity, 40-60%) with a 12 hr light/dark cycle and free access to water and food. The health status and physical activity of the rats were monitored every day. They were randomly divided into three groups (sham, IDD+PBS, IDD+PRP). Each group had 16 rats. As the lumbar spine of rats is more similar to that of humans in biomechanics, we used the lumbar spine to construct the rat IDD model (20). Similar to the previously described method, we used the annulus fibrosus needle puncture to build the IDD model (21). The rats were anesthetized with 1% pentobarbital (4 ml/kg, intraperitoneal injection). With rats in the supine position, a sagittal small skin incision was performed under aseptic techniques to expose the L4/5 and L5/6 for puncture. The discs were punctured with a 25-gauge sterile needle in a parallel direction to the endplates towards the NP with an angle of 30-60^°^ to the sagittal plane intervertebral disc. The needle was punctured to 2-3 mm depth, rotated 360^°^, and held in position for 30 sec. The puncture was not performed on the rats in the sham group. After suturing, the rats were put back into their cages. Four weeks later, all rats were anesthetized with 1% pentobarbital (4 ml/kg, intraperitoneal injection) again, L4/5 and L5/6 were exposed per the above methods, then 0.1 ml PBS and 0.1 ml PRP were injected into the IDD+PBS group and the IDD+PRP group by the above disc puncture, respectively. The sham group was not punctured. After the surgery, all rats were intraperitoneally injected with 8×104 U penicillin sodium salt once a day for three consecutive days to prevent infection. In addition to individual feeding for 24 hr after surgery, they had free access to water and food in the cage for the rest of the time. The rats were monitored regularly over the next few days to ensure no complications. The rats were sacrificed with an overdose of pentobarbital after another four weeks, and the NP tissues were extracted for subsequent experiments. All experimental protocols were approved by the Institutional Experimental Animal Ethics Committee of Nankai University (2023-SYDWLL-000353). Experiments complied with the Guidelines on the Humane Treatment of Laboratory Animals established by the Ministry of Science and Technology of the People’s Republic of China (Policy No. 2006 398). 

### Preparation of PRP

In the current study, we used allograft blood for PRP instead of autograft (22, 23). PRP was prepared using a double-spin centrifugation protocol (24). The healthy male SD rats were selected to be anesthetized with 1% pentobarbital (4 ml/kg, intraperitoneal injection). Firstly, 10 ml of whole blood was drawn from the abdominal aorta and centrifuged at 200×g for 10 min. This short step divided the blood into three layers: plasma, platelets, leucocytes (the “buffer coat”), and erythrocytes from top to bottom. Then, supernatants, plasma, and platelets in the superior layer were collected and centrifuged for another 10 min at 300×g. Lastly, the supernatant was discarded, and the remaining precipitates (approximately 1.5 ml) contributed to PRP. The sterile preparation kit (Regen Laboratories SA Inc., Switzerland) was used for human PRP preparation. Ten milliliters of whole blood from healthy volunteers were aspirated into a tube containing anticoagulant and centrifuged at 3,000×g for 15 min to produce 3-4 ml of PRP (buffy coat layer), which contained platelets, red blood cell-poor cells, and leukocyte-poor cells After preparation, 3 ml of supernatant PRP was aspirated and kept at –20 ^°^C until used.

### Human NP tissues isolation and culture

Human lumbar NP tissues were obtained from 36 patients with degenerative disc disease. The experimental protocol was approved by the Science and Technology Ethics Committee of TFCH (YC-BY-LC-2023-101), and all patients provided written informed consent. Used ophthalmic scissors to cut NP tissues into minced meat with a volume of about 1 mm^3^, followed by 0.25% trypsin digestion at 37 ^°^C for 2 hr and by 0.2% type II collagenase digestion at 37 ^°^C for 4 hr. After centrifugation, NP tissues were collected and cultured in a complete DMEM/F12 medium supplement with 15% bovine serum under the below condition: 5% CO_2, _100% humidity at 37 ^°^C in a constant temperature incubator. Since the degree of NP tissue degeneration differed in each patient, the third passage-NP cells were used to keep the nucleus pulposus cell in a steady state. The inflammatory factor concentrations may also decrease with the passage of NP cells. Thus, IL-1β (10 ng/ml) was used to induce inflammatory responses and establish a humanized IDD model per the previous report (25). Finally, these cells were randomly divided into the three groups of sham, IDD+PBS, and IDD+PRP (N=12, respectively). The expression of proteins related to the mTOR pathway was determined.

### Western blot

All protein levels related to the mTOR pathway (such as mTOR, p-mTOR, p70/S6K, p-p70/S6K, Akt, and p-Akt) were determined by Western blot and selected the glyceraldehyde-3-phosphate dehydrogenase (GAPDH) as an internal reference protein. After cutting the intervertebral disc into pieces, radioimmunoprecipitation (RIPA) lysis buffer was used to extract the total cellular protein; then, the protein was quantified by the bicinchoninic acid (BCA) method. Separated proteins to 20 μg per hole by 10% sodium dodecylsulphate polyacrylamide gel electrophoresis (SDS-PAGE), then transferred to polyvinylidene difluoride (PVDF) membranes. The current study used 5% non-fat milk with tris buffered saline with tween (TBST) to block the membranes for 2 hr. Then the membranes were incubated overnight at 4 ^°^C with different primary antibodies against mTOR, p70/S6K, p-p70/S6K, Akt (Abcam, ab8805, 1:1000), p-Akt (Abcam, ab81283, 1:500) and p-mTOR (Abcam, ab84400, 1:500). After washing the membranes three times with TBST, they were incubated with horse-radish peroxidase-conjugated secondary antibodies for onehour. The membranes were developed using an enhanced ECL kit (Beyotime, China). The densitometric quantification of the western blot bands was analyzed using Image J software (Bethesda, MD, U.S.A.). 

### Real-time polymerase chain reaction (RT-PCR)

RT-PCR was used to determine the expression of *aggrecan* and *collagen II* in the intervertebral disc of the rats. The total RNA of NP cells was extracted using the TRIzol method (Invitrogen, Carlsbad, CA, USA). Then, it was reversely transcripted to complementary deoxyribonucleic acid (cDNA) in a first-strand cDNA synthesis reaction with a One-Step PrimeScript RT-PCR kit (Takara Biotechnology, Dalian, China). According to the manufacturer’s protocol, the cDNA was amplified using Light Cycler (Roche Diagnostics) and SYBR Premix ExTaq (Takara). The 2-^ΔΔCt^ method evaluated expression fold changes with GAPDH/β-actin as the internal reference (26). The primer sequences were *aggrecan *of rat *(sense 5′-TGAAACCACCTCTGCATTCCA-3′; anti-sense 5′-GACGCCTCGCCTTCTTGAA-3′),* *collagen II* of rat* (sense 5′-GTCACAGAAGACCTCACGCCTC-3′; anti-sense 5′-TCCACACCGAATTCCTGCTC-3′), IL-1β* of rat *(sense 5′-GCAACTGTTCCTGAACTCAACT-3′; anti-sense 5′-ATCTTTTGGGGTCCGTCAACT-3′), IL-6* of rat (sense 5*′*-*TAGTCCTTCCTACCCCAATTTCC-3′; anti-sense 5′-TTGGTCCTTAGCCACTCCTTC-3′), TNF-α* of rat* (sense 5′-CCCTCACACTCAGATCATCTTCT-3′; anti-sense 5′-GCTACGACGTGGGCTACAG-3′), GAPDH *of rat (sense 5*′*-ACAACTTTGGTATCGTGGAAGG-3*′*; anti-sense 5*′*- *GCCATCACGCCACAGTTTC-3′), β-actin *of rat*  (sense 5′-TTTTGTGCCTTGATAGTTCGC-3′; anti-sense 5′-GAGTCCTTCTGACCCATACCC-3′), IL-1β* of human *(sense 5′-GCGGCCAGGATATAACTGACTTC-3′; anti-sense 5′-TCCACATTCAGCACAGGACTCTC-3′), IL-6* of human (sense *5′***-***ACTCACCTCTTCAGAACGAATTG-3′; anti-sense 5′-CCATCTTTGGAAGGTTCAGGTTG-3′), TNF-α* of human* (sense 5′-GAGGCCAAGCCCTGGTATG-3′; anti-sense 5′-CGGGCCGATTGATCTCAGC-3′)*, *GAPDH *of human *(sense 5′-TGAAGGTCGGAGTCAACGGATTTGGT-3′; anti-sense 5′-CATGTGGGCCATGAGGTCCACCAC-3′***), **and* β-actin *of human*  (sense 5′-CACCATTGGCAATGAGCGGTTC-3′; anti-sense 5′-AGGTCTTTGCGGATGTCCACGT -3′).*

### Measurement of oxidative stress

The activity of MDA, SOD, and GSH was respectively measured by MDA assay kit (Boxbio, China), SOD assay kit (Dojindo, Japan), and GSH assay kit (Beyotime, China) based on the spectrophotometric method according to the manufacturer’s instructions. 

### Statistical analysis

All quantitative data are presented as mean ± SD. Statistical analyses were performed using one-way analysis of variance (ANOVA) and Dunnett’s *post hoc* test by GraphPad Prism (USA). *P*<0.05 was considered statistically significant.

## Results

### PRP affected ECM degradation, inflammatory factors, oxidative stress, and apoptosis in IDD rats

**Figure 1:**
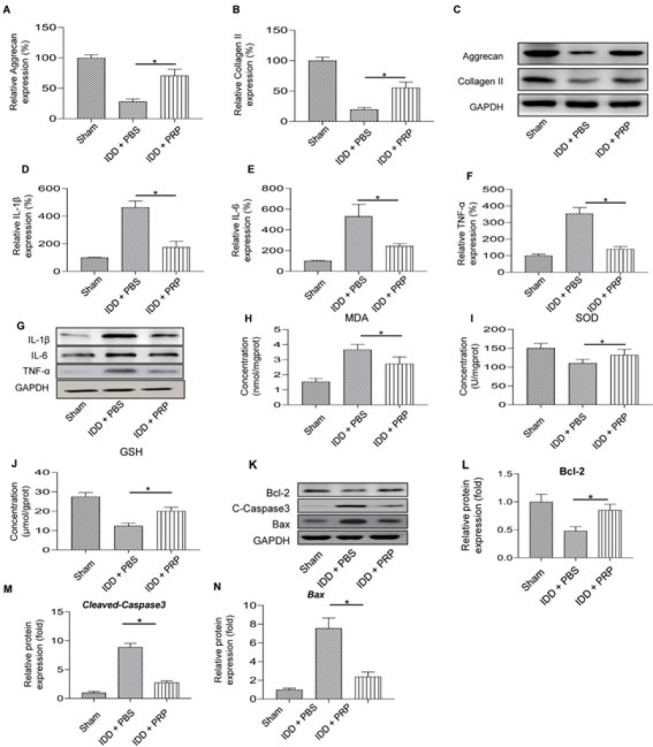
PRP affected ECM degradation, inflammatory factors, oxidative stress, and apoptosis in IDD rats

The rat IDD model was initially established to explore the efficacy of PRP. The aggrecan and collagen II expression levels were first measured by RT-PCR and western blot (Figure 1A-1C). Aggrecan and collagen II expression were significantly decreased in the IDD+PBS group than in the sham group but significantly promoted after PRP treatment in the IDD+PRP group (all *P*<0.05). The mRNA expression of *IL-1β*, *IL-6,* and *TNF-α* was higher than those in the sham group, and it decreased significantly in the IDD+PRP group (Figure 1D-1F, all *P*<0.05). Western blot validated the protein expression levels, showing a similar result with RT-PCR (Figure 1G). The expression of MDA, SOD, and GSH was detected using an assay kit (Figure 1H-1J). Compared with the IDD+PBS group, the expression of SOD and GSH was higher in IDD+PRP group (all *P*<0.05). On the contrary, the expression of MDA decreased in the IDD+PRP group (*P*<0.05). The apoptosis-associated proteins (Bcl-2, cleaved-Caspase 3, Bax) were also detected with western blot (Figure 1K). Anti-apoptosis expression of *Bcl-2* was down-regulated in the IDD+PBS group but reversely up-regulated in the IDD+PRP group (Figure 1L,* P*<0.05). The apoptosis-associated mRNA expression (*cleaved-Caspase 3*, *Bax*) presented the opposite results (Figure 1M-1N). These results revealed that PRP could affect ECM degradation, inflammatory factors, oxidative stress, and apoptosis in IDD rats.

### PRP inhibited the mTOR signaling pathway in IDD rats

**Figure 2:**
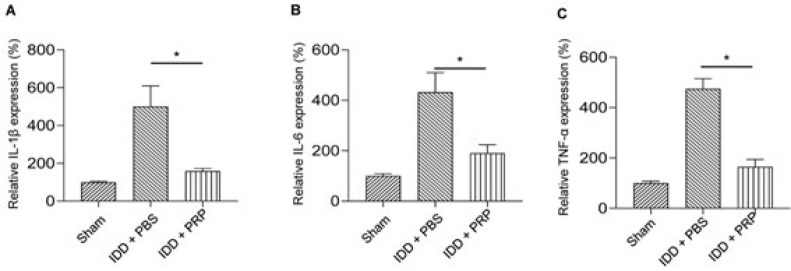
Relative protein expression of mTOR signaling pathway among the three groups of sham, IDD+PBS, and IDD+PRP

Western blot (Figure 2A) and Image J software were used to detect the expression levels of related proteins and phosphorylation in the mTOR signaling pathway. Compared with the IDD+PBS group, the expression of *p-mTOR *(Figure 2C), *p-p70/S6K* (Figure 2E), and *p-Akt* (Figure 2G) were much lower in the IDD + PRP group (all *P*<0.05). No significant differences in the expression of *mTOR, p70/S6K,* and *Akt *were found between the IDD+PBS group and the IDD+PRP group. Our results showed PRP could inhibit the mTOR signaling pathway in IDD rats.

### Expression of inflammatory factors in human NP cells treated with human PRP

**Figure 3:**
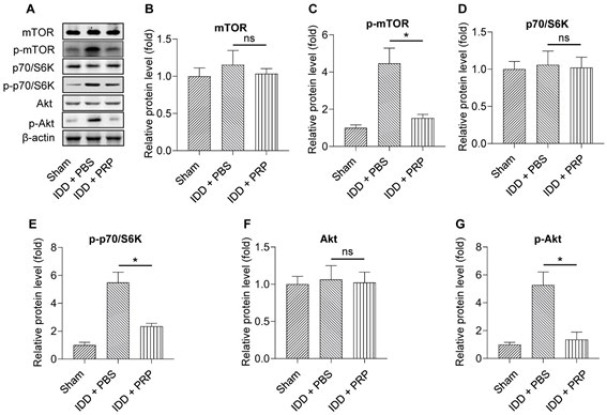
Relative IL-1β, IL-6, and TNF-α expression among the three groups of sham, IDD+PBS, and IDD+PRP

We also established a humanized IDD model in human lumbar NP cells and treated it with human PRP. Compared with the sham group, IL-1β, IL-6, and TNF-α expression in the IDD+PBS group was higher, but it decreased significantly after PRP treatment (the IDD+PRP group) (Figure 3). This result showed inflammatory mediators and signaling pathways were involved in human IDD. 

### PRP inhibited the mTOR signaling pathway in human NP cells

**Figure 4:**
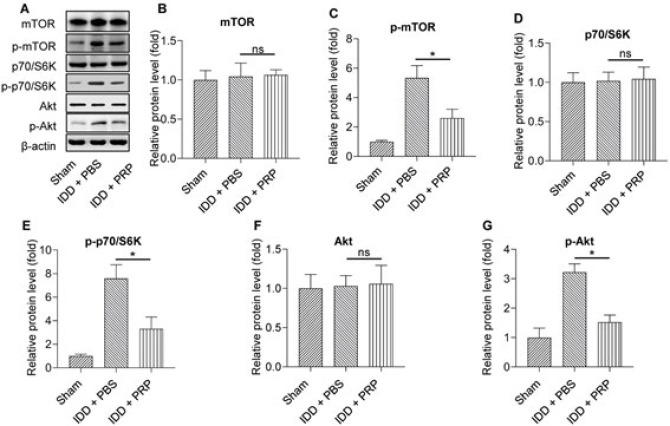
Relative protein expression of the mTOR signaling pathway in the three group of sham, IDD+PBS, and IDD+PRP

Then, we investigated the inhibition of PRP to the mTOR signaling pathway. Western blot (Figure 4A) and Image J software were used to detect the expression levels of related proteins and phosphorylation in the mTOR signaling pathway. No significant differences existed in the mTOR, p70/S6K, and Akt levels between the IDD+PBS group and IDD+PRP group. After PRP treatment, *p-mTOR* (Figure 4C), *p-p70/S6K* (Figure 4E), and *p-Akt* (Figure 4G) levels decreased significantly (all *P*<0.05). These results showed that PRP could inhibit the mTOR signaling pathway.

## Discussion

Several *in vitro* and *in vivo *(animal) studies have shown that PRP has significant biological efficacy in stimulating IVD cells to repair tissue and treat discogenic LBP caused by IDD (16). However, the mechanism is still unclear. We investigated PRP’s efficacy and the potential mechanisms for treating IDD through *in* *vivo* (animal) experiments. PRP could decrease oxidative stress and the expression levels of inflammatory factors in NP cells of IDD rats. What’s more, PRP inhibited ECM degradation and apoptosis in IDD rats. The above efficacy may be achieved by regulating the Akt/mTOR/p70S6K signaling pathway. In addition, an *in vitro* experiment was also conducted. Our findings showed that PRP inhibited the expression of inflammatory factors and the mTOR signaling pathway in human NP cells. These results provided insights into PRP therapy and mTOR signaling pathway intervention in treating IDD.

Inflammatory factors have been shown to be associated with IDD (4, 27, 28). The progression of IDD is accompanied by elevated levels of various pro-inflammatory cytokines, including IL-1α, IL-1β, IL-6, IL-17, and TNF-α. Among them, IL-1β and TNF-α are identified as the most important pro-inflammatory cytokines (4). With powerful pro-inflammatory activities, they can promote the secretion of various pro-inflammatory mediators. Moreover, IL-1β and TNF-α are positively related to pain intensity (29). The current study found that IL-1β, IL-6, and TNF-α expression levels decreased in IDD rats after PRP treatment. Similarly, a clinical trial reported PRP was an effective therapy in treating rheumatoid arthritis patients through its down-regulating effect on inflammatory cytokines, such as IL-1β and TNF α (30). Thus, there are reasons to believe PRP can improve local joint inflammation, disease activity, and quality of life. 

The imbalance of ECM homeostasis is closely related to the pathological process of IDD (31). ECM degradation will impair the biomechanical characteristic of NP, exacerbating Disc degeneration. ECM homeostasis is maintained by the balance between catabolic enzymes, MMPs and a disintegrin, and metalloproteinases with thrombospondin motifs (ADAMTSs), and their anti-catabolic inhibitors, tissue inhibitors of metalloproteinases (TIMPs) (32). Increased MMPs and ADAMTSs relative to TIMPs are often observed in human IVD specimens (33) and multiple animal models of degenerative discs (34-36). The decrease of aggrecan and collagens II resulted in degraded matrix components (37). We found PRP could improve the expression of aggrecan and collagen II in IDD rats. It was also indicated that the PRP *in* *vitro* increased the production of the major matrix components (type II collagen and aggrecan) in porcine IDD (38). All the above results suggest that PRP can inhibit ECM degradation in IVD cells, thereby protecting the IVD structure. 

Oxidative stress and subsequent apoptosis of NP cells are also important contributors to the development of IDD. Oxidative stress can accelerate IVD degeneration through multiple signaling pathways, such as the NF-κB pathway, MAPK pathway, and PI3K/Akt pathway (9). MDA is a well-established biomarker of oxidative stress, whereas SOD and GSH reflect levels of antioxidant substances. We detected increased SOD and GSH in IDD rats after injecting PRP. A decreased oxidative stress was found in the current study. A previous study reported PRP could elevate the GSH level in rat models of osteoarthritis (39). These data all confirmed the antioxidant properties of PRP. Disc cell decline is another major feature of IDD, mainly caused by apoptotic cell death (40). The balance between Bax and Bcl-2 can influence the proapoptotic pathway. The current study found PRP could decrease apoptosis-related protein (cleaved-caspase 3 and Bax) and increase Bcl-2. PRP can prevent glucocorticoid-induced apoptosis in a rat model of osteonecrosis of the femoral head by promoting Bcl-2 expression. Some studies have also revealed PRP can prevent apoptosis in animal models (41, 42). Thus, our findings suggested that PRP may be used to treat IDD by inhibiting apoptosis. 

Autophagy is another mechanism that leads to IDD. Compared with the healthy disc, IDD has an up-regulated expression of autophagy-related genes (43). As a serine/threonine protein kinase, mTOR was reported to be a key negative regulatory modulator in upstream autophagy. The increased vascular endothelial growth factor expression in IVD tissues can activate the mTOR signaling pathway, inhibiting autophagy and accelerating IDD (44). mTOR exists in two complexes: one is called mTOR complex 1 (mTORC1), which contains the regulatory-associated protein of mTOR (RAPTOR), and another is mTOR complex 2 (mTORC2), containing the rapamycin-insensitive companion of mTOR (RICTOR) (45). mTORC1 can regulate p70/ribosomal S6 kinase (p70/S6K) and negatively regulate autophagy by the upstream Akt (45). The role of mTORC2 is still unclear; however, mTORC2 was reported to regulate Akt (46). Furthermore, phosphorylation of *Akt* was associated with *PI3K* and *mTOR*. In a word, the mTOR plays an important role in the process of autophagy. 

PRP is an autologous human plasma preparation with increased platelet concentration. It contains a high level of growth factors and mediators. Because of its potential ability to repair tissue, PRP is increasingly accepted for various musculoskeletal disorders. Therefore, PRP also has potential efficacy in IDD. A review article showed that PRP effectively stimulated IVD cell proliferation and ECM metabolism (16). However, the mechanism is still unclear. We found the mTOR signaling pathway was involved in the process of PRP improving IDD in rats. PRP would inhibit the expression of p-mTOR, p-p70/S6K, and p-Akt. Although the Akt/mTOR/p70S6K signaling pathway has not been reported in PRP-treated IDD, a study demonstrated that PRP could play a unique role in protecting anterior cruciate ligament fibroblasts through regulation of PI3K/Akt/mTOR signaling pathways (47). Finally, we also established a humanized IDD model with IL-1β. And PRP was used to treat the humanized IDD. Similarly, the expression levels of inflammatory factors in human NP cells were significantly reduced. And the mTOR pathway was inhibited. The latest review article also revealed the role of the mTOR signaling pathway in IDD. 

There are still some limitations in the current study. First, the rat IDD model used in the present study could not reflect the natural course of human IDD. Second, tissue section staining, IHC, and MRI were not used to assess degeneration and repair after treatment. Third, the experiment was designed with insufficient consideration, and the blood tissue of patients was not reserved. Thus, PRP data* in vivo *of humans and some clinical indicators were lacking in the current study. Last, only inflammatory factors were analyzed in the humanized IDD model treated with PRP. Thus, more translational research is critically needed to maximize the clinical potential of PRP in IDD, as we saw a positive result in the current study. 

A. Expression of *aggrecan* increased in the IDD+PRP group. B. Expression of *collagen II* increased in the IDD+PRP group. C. Proteinic expression of aggrecan and collagen II. D. Expression of *IL-1β* decreased in the IDD+PRP group. E. Expression of *IL-6* decreased in the IDD+PRP group. F. Expression of *TNF-α* decreased in the IDD+PRP group. G. Proteinic expression of *IL-1β, IL-6*, and *TNF-α*. H. Expression of MDA decreased in the IDD+PRP group. I. Expression of SOD increased in the IDD+PRP group. J. Expression of GSH increased in the IDD+PRP group. K. Proteinic expression of *Bcl-2*, *Cleaved-Caspase3*, and *Bax*. L. Expression of Bcl-2 increased in the IDD+PRP group. M. Expression of Cleaved-Caspase3 decreased in the IDD+PRP group. N. Expression of Bax decreased in the IDD+PRP group

All values are presented as mean±SD.*P<0.05

ECM: Extracellular matrix; IDD, Intervertebral disc degeneration; PRP, Platelet-rich plasma

A. Proteinic expression of the *mTOR* signaling pathway. B. Expression of *mTOR* was similar in the three groups. C. Expression of *p-mTOR* decreased in the IDD+PRP group. D. Expression of *p70/S6K* was similar in the three groups. E. Expression of *p-p70/S6K* decreased in the IDD+PRP group. F. Expression of *Akt* was similar in the three groups. G. Expression of *p-Akt* decreased in the IDD+PRP group

The rat IDD models were established. All values are presented as mean±SD. *P<0.05

IDD: Intervertebral disc degeneration; PRP: Platelet-rich plasma

A. Expression of *IL-1β* decreased in the IDD + PRP group. B. Expression of *IL-6* decreased in the IDD+PRP group. C. Expression of *TNF-α* decreased in the IDD+PRP group

The IDD models were established in human lumbar nucleus pulposus cells.

All values are presented as mean±SD. *P<0.05

IDD: Intervertebral disc degeneration; PRP: Platelet-rich plasma

A. Proteinic expression of the *mTOR* signaling pathway. B. Expression of *mTOR* was similar in the three groups. C. Expression of *p-mTOR* decreased in the IDD+PRP group. D. Expression of *p70/S6K* was similar in the three groups. E. Expression of *p-p70/S6K* decreased in the IDD+PRP group. F. Expression of *Akt* was similar in the three groups. G. Expression of *p-Akt* decreased in the IDD+PRP group.

The IDD models were established in human lumbar nucleus pulposus cells

All values are presented as mean±SD. *P<0.05

IDD: Intervertebral disc degeneration; PRP: Platelet-rich plasma

## Conclusion

PRP may be a potential therapy for IDD via the mTOR signaling pathway in regulating and affecting ECM degradation, inflammatory factors, oxidative stress, and apoptosis.

## Data Availability

Datasets are available from the corresponding author upon reasonable request.

## Declaration

We declare “We have not used any AI tools or technologies to prepare this manuscript
